# Adjuvanted recombinant zoster vaccine in adult autologous stem cell transplant recipients: polyfunctional immune responses and lessons for clinical practice

**DOI:** 10.1080/21645515.2021.1953346

**Published:** 2021-08-18

**Authors:** Edward A. Stadtmauer, Keith M. Sullivan, Mohamed El Idrissi, Bruno Salaun, Aránzazu Alonso Alonso, Charalambos Andreadis, Veli-Jukka Anttila, Adrian JC Bloor, Raewyn Broady, Claudia Cellini, Antonio Cuneo, Alemnew F. Dagnew, Emmanuel Di Paolo, HyeonSeok Eom, Ana Pilar González-Rodríguez, Andrew Grigg, Andreas Guenther, Thomas C. Heineman, Isidro Jarque, Jae-Yong Kwak, Alessandro Lucchesi, Lidia Oostvogels, Marta Polo Zarzuela, Anne E. Schuind, Thomas C. Shea, Ulla Marjatta Sinisalo, Filiz Vural, Lucrecia Yáñez San Segundo, Pierre Zachée, Adriana Bastidas

**Affiliations:** a University of Pennsylvania, Philadelphia, PA, USA; b Division of Hematologic Malignancies and Cellular Therapy, Duke University Medical Center, Durham, NC, USA; c GSK, Rue de l’Institut 89, Rixensart, Belgium; d Hospital Quirón Madrid, Pozuelo de Alarcón, Spain; e University of California Medical Center, San Francisco, CA, USA; f Inflammation Center, University of Helsinki and Helsinki University Hospital, Helsinki, Finland; g Haematology and Transplant Unit, The Christie NHS Foundation Trust, Manchester, UK; h Vancouver General Hospital, Vancouver, BC, Canada; i U.O. di Ematologia, Ospedale Santa Maria Delle Croci, Ravenna, Italy; j Unità Operativa di Ematologia, Azienda Osp. Universitaria Arcispedale S. Anna, Cona, Italy; k GSK, Rockville, MD, USA; l National Cancer Center, Goyang-si, Republic of Korea; m Hospital Universitario Central de Asturias, Oviedo, Spain; n Department of Clinical Haematology, Austin Health, Heidelberg, Australia; o Universitaetsklinikum Schleswig-Holstein, Kiel, Germany; p Halozyme Therapeutics, San Diego, CA, USA; q Hematology Department & CIBERONC, Instituto Carlos III, Hospital Universitario y Politécnico la fe, Valencia, Spain; r Chonbuk National University Hospital, DukJin-Gu, Republic of Korea; s Hematology Unit, IRCCS Istituto Romagnolo per lo Studio dei Tumori (IRST) “Dino Amadori”, Meldola, Italy; t CureVac AG, Tübingen, Germany; u Hospital Clínico San Carlos, Madrid, Spain; v Division of Hematology and Medical Oncology, University of North Carolina at Chapel Hill, Chapel Hill, NC, USA; w Hematology Unit, Department of Internal Medicine, Tampere University Hospital, Tampere, Finland; x Ege University Medical Faculty Hospital, Izmir, Turkey; y Hematology Department, Hospital Universitario Marqués De Valdecilla-IDIVAL, University of Cantabria, Santander, Spain; z Hematologie - Oncologie, Ziekenhuisnetwerk Antwerpen - ZNA Stuivenberg & ZNA Middelheim, Antwerpen, Belgium; aa GSK, Wavre, Belgium

**Keywords:** Autologous hematopoietic stem cell transplant, cell-mediated immunity, polyfunctionality, humoral immune response, adjuvanted recombinant zoster vaccine, vaccine efficacy

## Abstract

Immunocompromised individuals, particularly autologous hematopoietic stem cell transplant (auHSCT) recipients, are at high risk for herpes zoster (HZ). We provide an in-depth description of humoral and cell-mediated immune (CMI) responses by age (protocol-defined) or underlying disease (post-hoc) as well as efficacy by underlying disease (post-hoc) of the adjuvanted recombinant zoster vaccine (RZV) in a randomized observer-blind phase III trial (ZOE-HSCT, NCT01610414). 1846 adult auHSCT recipients were randomized to receive a first dose of either RZV or placebo 50–70 days post-auHSCT, followed by the second dose at 1–2 months (M) later. In cohorts of 114–1721 participants, at 1 M post-second vaccine dose: Anti-gE antibody geometric mean concentrations (GMCs) and median gE-specific CD4[2+] T-cell frequencies (CD4 T cells expressing ≥2 of four assessed activation markers) were similar between 18–49 and ≥50-year-olds. Despite lower anti-gE antibody GMCs in non-Hodgkin B-cell lymphoma (NHBCL) patients, CD4[2+] T-cell frequencies were similar between NHBCL and other underlying diseases. The proportion of polyfunctional CD4 T cells increased over time, accounting for 79.6% of gE-specific CD4 T cells at 24 M post-dose two. Vaccine efficacy against HZ ranged between 42.5% and 82.5% across underlying diseases and was statistically significant in NHBCL and multiple myeloma patients. In conclusion, two RZV doses administered early post-auHSCT induced robust, persistent, and polyfunctional gE-specific immune responses. Efficacy against HZ was also high in NHBCL patients despite the lower humoral response.

## PLAIN LANGUAGE SUMMARY

What is the context?

- After haematopoietic stem cell transplantation, patients have impaired immunity from conditioning chemotherapy regimens, often exacerbated by underlying diseases, putting them at high risk of developing herpes zoster. In this population, antiviral prophylaxis is the current standard of care to reduce herpes zoster risk. Vaccination provides an additional means to prevent herpes zoster. Live-attenuated vaccines are generally contraindicated in immunocompromised patients. A non-live, adjuvanted recombinant zoster vaccine (RZV, Shingrix, GSK), has been approved for use in adults ≥50 years of age in the European Union, United States, Canada, Australia, Japan, and China. This vaccine is highly efficacious at preventing herpes zoster in adults over 50 years of age, as demonstrated in large, placebo-controlled randomised trials. Importantly, Shingrix use is not contraindicated in immunocompromised conditions, and was found to be highly efficacious in adults who had recently undergone autologous haematopoietic stem cell transplant.

What is new?

- In autologous haematopoietic stem cell transplant recipients in whom Shingrix has demonstrated efficacy, two doses elicited high and persistent immune responses. Date presented here further support our understanding of the impact of specific factors such as age or underlying diseases on the vaccine’s effect in the population studied, as well as the characteristics of the elicited cell-mediated immune responses.

What is the impact?

- These results indicate that Shingrix, given shortly after haematopoietic stem cell transplant, can induce robust immune responses and reduce the risk of herpes zoster, even in individuals with immunosuppression due to underlying disease and/or use of immunosuppressive therapies, regardless of age or underlying disease.

## Introduction

Herpes zoster (HZ) develops following the reactivation of latent varicella-zoster virus (VZV), particularly in individuals with reduced immune function, such as older adults, and in persons with acquired immunodeficiency or receiving immunosuppressive therapies.^1,2^ It also commonly occurs in patients who have undergone allogeneic, syngeneic, or autologous hematopoietic stem cell transplant (HSCT), in whom T-cell immunity is diminished (incidence range: 16–30%).^3–6^

To prevent virus-associated infections, including HZ, antiviral prophylaxis is commonly administered to patients after HSCT.^7,8^ However, the efficacy of the prophylaxis is impacted by the adherence to medication and its duration and HZ risk increases once prophylaxis has been stopped.^8–10^

Improved prevention strategies to provide sustained protection against HZ are therefore needed.^8^ Vaccination can provide long-term protection, but live-attenuated vaccines are contraindicated in immunocompromised individuals due to the risk of disseminated disease.^11,12^

An adjuvanted recombinant zoster vaccine (RZV, *Shingrix*, GSK) demonstrated >90% vaccine efficacy against HZ in phase III clinical trials conducted in participants aged ≥50 and ≥70 years,^13,14^ and was first licensed in 2017 for use in adults aged ≥50 years.^15^ Another study showed that immune responses to RZV persist at least ten years in adults vaccinated at age ≥60 years and modeling predicts that these will persist for up to 20 years.^16^ As this is a non-live, recombinant subunit vaccine, there is no risk of it causing disseminated HZ in immunocompromised individuals, and RZV could represent an important strategy to prevent HZ in HSCT patients.

Due to diminished immunity following HSCT conditioning regimens, or due to the underlying malignancy, patients may be unable to mount an adequate protective immune response to vaccination given shortly after transplantation.^17^ Nevertheless, autologous HSCT patients aged ≥18 years showed strong glycoprotein E (gE)-specific humoral and CMI responses to RZV in a phase I/IIa study and a phase III efficacy study (ZOE-HSCT).^18,19^ In ZOE-HSCT, RZV administered 50–70 days post-transplant, demonstrated 68% efficacy (95% confidence interval [CI] 56–78) in preventing HZ and induced robust gE-specific humoral and CMI responses.^19^ Here we further characterize gE-specific humoral and CMI responses by age and underlying diseases, and also present vaccine efficacy according to underlying diseases in the ZOE-HSCT study population.

## Materials and methods

### Study design

This was a randomized, observer-blind, placebo-controlled, parallel-group phase III study, conducted in 167 centers in 28 countries (NCT01610414). The study protocol was reviewed and approved by relevant Institutional Review Boards or Independent Ethics Committees. The full study protocol is available as part of the primary publication.^19^

### Participants

Eligible adults aged ≥18 years had undergone autologous HSCT 50–70 days before the first dose of study vaccine. Detailed inclusion and exclusion criteria have been described previously.^19^ Before study start, all participants provided written informed consent.

### Randomization and masking

All eligible participants were randomized 1:1 to receive either RZV or placebo using a minimization procedure, which has been previously described.^19^ At designated centers, participants were further randomly allocated to the humoral and CMI sub-cohorts until the sub-cohort targets were reached. The CMI sub-cohort included participants from Belgium, France, Japan, Spain, and the United States, who were enrolled at centers that had access within 24 hours from collection time to a peripheral blood mononuclear cell processing facility validated by GSK. The humoral immunity sub-cohort included participants from both the CMI sub-cohort and additional participants from designated centers in Australia, Canada, and Korea.

Vaccine/placebo doses were administered by unmasked study staff who did not participate in the study assessments. Further details on blinding undertaken for the assessment of efficacy have been presented previously.^19^

### Procedures

Each 0.5 ml RZV dose contained recombinant VZV gE (50 μg) and the AS01_B_ adjuvant system (containing MPL, QS21, and liposome).^15^ Placebo contained lyophilized sucrose reconstituted in 0.9% saline solution. The first dose was administered 50–70 days post-transplantation and the second dose one to two months (M) thereafter.

### Outcomes

Vaccine efficacy against HZ (primary study outcome), HZ complications, and HZ-related hospitalizations, as well as safety and overall immunogenicity results, have been described previously.^19^

Here we present humoral and CMI responses by age (18–49 years and ≥50 years), which were assessed in protocol-defined sub-group analyses. Vaccine efficacy against HZ and immunogenicity of RZV by underlying disease (multiple myeloma [MM], non-Hodgkin B-cell and T-cell lymphoma [NHBCL, NHTCL], Hodgkin lymphoma [HL], acute myeloid leukemia [AML], and solid malignancies and others), as well as polyfunctional gE-specific CD4 T-cell responses were assessed post-hoc.

### Assessments

Assessment of HZ cases and estimation of vaccine efficacy have been described in detail previously (Supplementary Text 1).^19^

Blood samples (8 ml) were collected from all participants at pre-vaccination and 1 M post-dose two to contribute to the correlate of protection assessment (not reported here). These blood samples were used to evaluate humoral immunogenicity according to underlying diseases, which are described herein. Additional blood samples (8 ml) were taken from the humoral immunogenicity sub-cohort at 1 M post-dose one, and at 12 M and 24 M post-dose two. Participants in the CMI sub-cohort provided additional 30 ml blood samples at all five predefined study visits.

Anti-glycoprotein E (gE) antibody concentrations were measured using a previously described assay.^20^ The frequency of gE-specific CD4 T cells expressing at least two of the following activation markers (CD4[2+] T cells) per 10^6^ CD4 T cells was calculated: interferon-γ (IFN-γ), interleukin-2 (IL-2), tumor necrosis factor-α (TNF-α), and CD40 ligand (CD40L). For the analysis of gE-specific T-cell polyfunctionality, the frequency of CD4 T cells expressing one or any combination of two, three, or four of the four activation markers assessed was calculated. Additional details have been disclosed previously,^20^ and are as described in Supplementary Text 2.

### Statistical analysis

All endpoints presented here are descriptive. Therefore, no formal sample size calculations were performed for these analyses.

Post-hoc vaccine efficacy evaluation and analyses of humoral immunity according to underlying diseases were performed on the modified total vaccinated cohort, which included all participants who received both doses and did not develop HZ before 1M post-dose two. Humoral immune responses according to underlying diseases were evaluated in the modified total vaccinated cohort (from samples collected for the correlate of protection assessment, which is not reported here) rather than in the according-to-protocol immunogenicity sub-cohort to provide a greater sample size in each subgroup.

Humoral immune responses per age and CMI responses per age and underlying diseases were assessed in the according-to-protocol immunogenicity cohorts, which included all eligible participants from the humoral and CMI sub-cohorts, respectively, who received both doses, complied with the protocol and had available immunogenicity endpoint measurements.

Anti-gE antibody geometric mean concentrations (GMCs) and their 95% CIs were determined. The vaccine response in terms of anti-gE antibody concentration (i.e., humoral vaccine response) was defined as a ≥4-fold increase in the anti-gE antibody concentration compared either with the pre-vaccination concentration (initially seropositive participants) or with the anti-gE antibody cutoff value for seropositivity (97 milli-International Units per milliliter [mIU/mL]) (initially seronegative participants).

The vaccine response in terms of CD4[2+] T cell frequency (i.e., CMI vaccine response) was defined as a ≥2-fold increase in the frequency of CD4[2+] T cells, as compared to pre-vaccination frequency (for participants with pre-vaccination CD4[2+] T-cell frequency ≥320 per 10^6^ CD4 T cells counted) or the cutoff (for participants with pre-vaccination frequencies below the cutoff).

Exact 95%CIs were computed at each time point for the humoral and CMI vaccine response rate using the Clopper Pearson exact method. The 95%CI for the GMCs was computed by anti-log transformation of the 95%CI for the mean of log-transformed concentrations.

The statistical analyses were performed using SAS Drug Development.

## Results

### Study population

**Figure 1.**
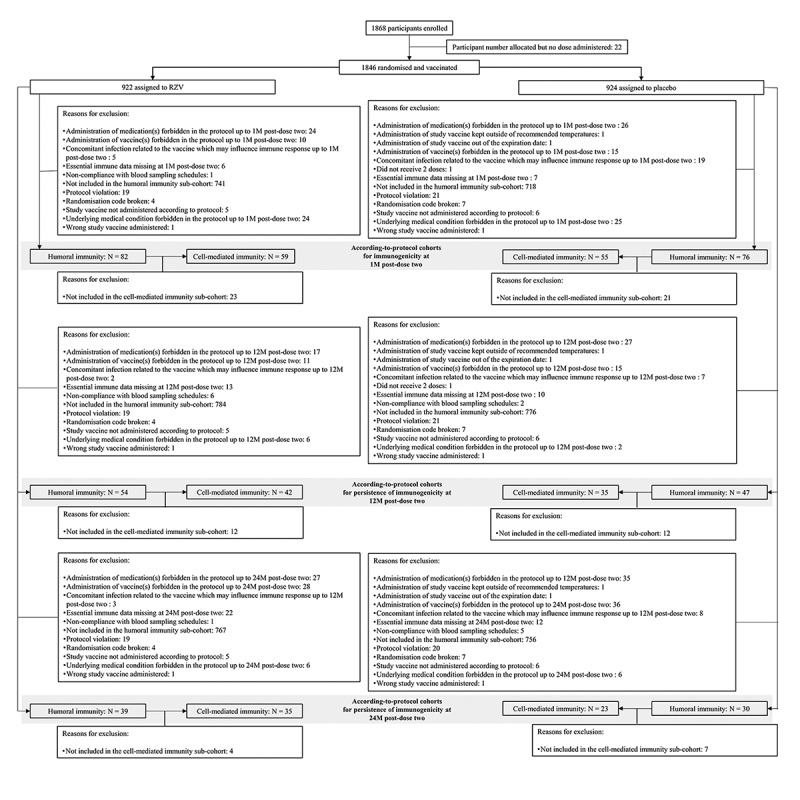
Trial profile. M = month; N = number of participants; RZV = adjuvanted recombinant zoster vaccine.

Characteristics of the overall study population, as well as reasons for withdrawals from the modified total vaccinated cohort have been previously published.^19^ Of the 1846 participants (RZV: 922, placebo: 924) vaccinated between 13 July 2012 and 31 July 2015, 1721 (RZV: 870, placebo: 851) were included in the modified total vaccinated cohort and 158 (RZV: 82, placebo: 76) were included in according-to-protocol cohort for humoral immunogenicity at 1M post-dose two, of whom 114 (RZV: 59, placebo: 55) were also included in the according-to-protocol cohort for CMI at 1M post-dose two (Figure 1).

The demographic characteristics were balanced between the RZV and placebo groups in all cohorts evaluated (Table 1) and comparable to the TVC.^19^ Most participants were white, male, and aged ≥50 years, and MM and NHBCL were the predominant underlying diseases.

**Table 1.** Baseline demographic characteristics (modified total vaccinated cohort and according-to-protocol cohorts for humoral and cell-mediated immunogenicity)

	Modified total vaccinated cohort, N = 1721		According-to-protocol cohort			
			Humoral immunity sub-cohort, N = 158		Cell-mediated immunity sub-cohort, N = 114	
Characteristic	RZV (N = 870)	Placebo (N = 851)	RZV (N = 82)	Placebo (N = 76)	RZV (N = 59)	Placebo (N = 55)
**Age (years)**						
Mean ± SD	54.9 ± 11.5	55.1 ± 11.3	54.2 ± 11.8	56.5 ± 9.9	54.2 ± 10.5	55.6 ± 9.9
Age group-no. (%)						
18–49 years	213 (24.5)	212 (24.9)	26 (31.7)	17 (22.4)	20 (33.9)	14 (25.4)
≥50 years	657 (75.5)	639 (75.1)	56 (68.3)	59 (77.6)	39 (66.1)	41 (74.6)
**Gender-no. (%)**						
Female	323 (37.1)	317 (37.3)	29 (35.4)	29 (38.2)	18 (30.5)	23 (41.8)
Male	547 (62.9)	534 (62.7)	53 (64.6)	47 (61.8)	41 (69.5)	32 (58.2)
**Race-no. (%)**						
White	686 (78.9)	666 (78.3)	60 (73.2)	58 (76.3)	48 (81.4)	47 (85.5)
Black	15 (1.7)	23 (2.7)	3 (3.7)	1 (1.3)	3 (5.1)	1 (1.8)
Asian	138 (15.9)	139 (16.3)	17 (20.7)	16 (21.1)	6 (10.2)	6 (10.9)
Other	31 (3.6)	23 (2.7)	2 (2.4)	1 (1.3)	2 (3.4)	1 (1.8)
**Underlying disease-no. (%)**						
Multiple myeloma	472 (54.3)	465 (54.6)	44 (53.7)	42 (55.3)	33 (55.9)	32 (58.2)
Other diseases	398 (45.7)	386 (45.4)	38 (46.3)	34 (44.7)	26 (44.1)	23 (41.8)
Non-Hodgkin B-cell lymphoma	237 (27.2)	244 (28.7)	.	.	16 (27.1)	12 (21.8)
Non-Hodgkin T-cell lymphoma	43 (4.9)	40 (4.7)	.	.	3 (5.1)	3 (5.5)
Hodgkin lymphoma	74 (8.5)	60 (7.1)	.	.	1 (1.7)	3 (5.5)
Acute myeloid leukemia	20 (2.3)	16 (1.9)	.	.	2 (3.4)	2 (3.6)
Solid malignancies and others	24 (2.8)	26 (3.1)	.	.	4 (6.8)	3 (5.5)
Other hematologic malignancies	10	9	.	.	1	2
Amyloidosis	7	7	.	.	1	0
Solid malignancies	6	6	.	.	2	0
Systemic sclerosis	1	3	.	.	0	1
Multiple sclerosis	0	1	.	.	0	0

N = number of participants included in each group; no. (%) = number (percentage) of participants in each category; SD = standard deviation; RZV = adjuvanted recombinant zoster vaccine.

### Immune responses according to age

**Figure 2.**
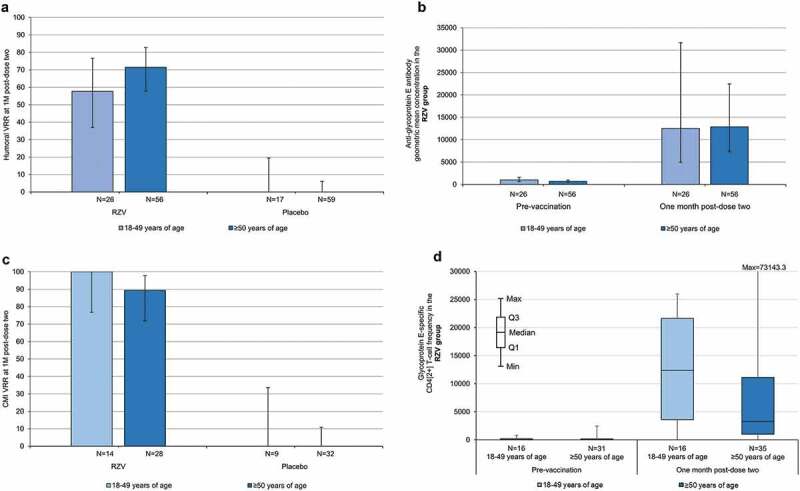
Humoral and cell-mediated immune responses according to age (according-to-protocol cohort for humoral immunogenicity). CMI = cell-mediated immunity; M = month; N = number of participants with available results; Q1, Q3 = first and third quartiles; RZV = adjuvanted recombinant zoster vaccine; In panels A–C, error bars depict two-sided exact 95% confidence intervals.

Before vaccination, ≥82.4% of participants were seropositive for anti-gE antibody across age cohorts and study groups. In RZV group participants aged 18–49 and ≥50 years, respectively, the humoral vaccine response rate was 57.7% (95%CI 36.9–76.6) and 71.4% (57.8–82.7) at 1 M post-dose two and 23.1% (5.0–53.8) and 56.0% (34.9–75.6) at 24 M post-dose two. In placebo group participants, the humoral vaccine response rate was ≤22.2% across age cohorts and time points. In RZV group participants aged 18–49 and ≥50 years, respectively, anti-gE antibody GMCs were 1011.0 (95%CI 629.7–1623.2) and 669.3 (460.2–973.5) mIU/mL at pre-vaccination, 12523.4 (4950.7–31679.7) and 12861.3 (7366.4–22455.0) mIU/mL at 1 M post-dose two, and 1492.5 (466.3–4777.1) and 4025.0 (1597.6–10140.1) mIU/mL at 24 M post-dose two. In the placebo group, post-vaccination anti-gE antibody GMCs remained at pre-vaccination levels in both age cohorts (Figure 2(a,b), Supplementary Table 1).

In RZV group participants aged 18–49 and ≥50 years, respectively, the CMI vaccine response rate was 100% (95%CI 76.8–100) and 89.3% (71.8–97.7) at 1 M post-dose two, and 100% (59.0–100) and 58.8% (32.9–81.6) at 24 M post-dose two. In placebo group participants, the CMI vaccine response rate was ≤20.0% across age cohorts and time points. In RZV group participants aged 18–49 and ≥50 years, respectively, median CD4[2+] T-cell frequencies were 77.9 (interquartile range: 9.7–213.9) and 34.0 (1.0–185.2) at pre-vaccination, 12365.5 (3591.1–21624.6) and 3294.2 (1017.1–11135.7) at 1 M post-dose two, and 3466.0 (1969.9–5087.5) and 1519.6 (281.4–3155.0) at 24 M post-dose two. In both age cohorts of the placebo group, median CD4[2+] T-cell frequencies remained near pre-vaccination levels up to and including the last assessment (Figure 2(c,2d), Supplementary Table 2).

### Immune responses according to underlying diseases

**Figure 3.**
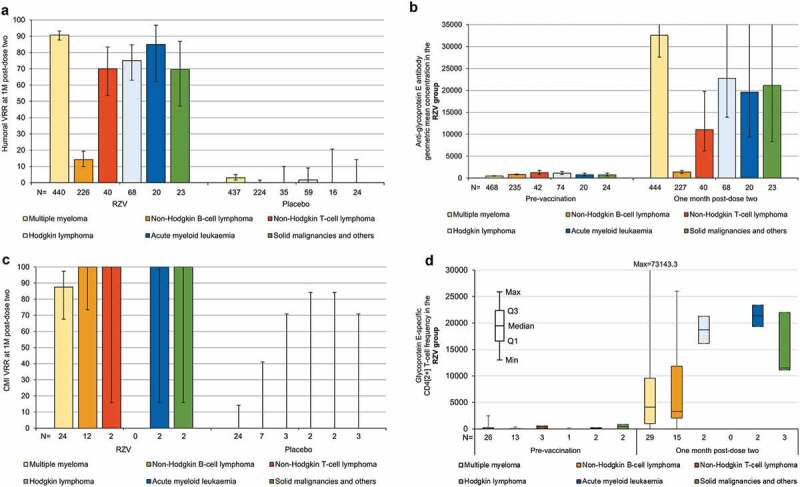
Humoral and cell-mediated immune responses according to underlying diseases (modified total vaccinated cohort [panels A and B] and according-to-protocol cohort for cell-mediated immunity [panels C and D], respectively). CMI = cell-mediated immunity; M = month; N = number of participants with available results; Q1, Q3 = first and third quartiles; RZV = adjuvanted recombinant zoster vaccine; In panels A–C, error bars depict two-sided exact 95% confidence intervals; in panel B, the 95% confidence interval upper limits at one month post-dose two are 38405.7 for multiple myeloma, 37353.5 for Hodgkin lymphoma, 40607.5 for acute myeloid leukemia, and 53708.3 for solid malignancies and others.

In the RZV group, the humoral vaccine response rate at 1 M post-dose two was lower among NHBCL patients (14.2%, 95%CI 9.9–19.4) compared to patients with any of the other underlying diseases (≥69.6%). In the placebo group, the humoral vaccine response rate was ≤3.0% across underlying diseases at 1 M post-dose two. In the RZV group, anti-gE antibody GMCs increased substantially (i.e., >9-fold) at 1 M post-dose two compared to pre-vaccination irrespective of the underlying disease with the exception of NHBCL (<2-fold increase). No increases were observed in placebo recipients (Figure 3(a,b), Supplementary Table 3).

Due to the relatively limited size of the CMI sub-cohort, at each time point, few participants with underlying diseases other than MM or NHBCL had available data. In the RZV group, the CMI vaccine response rate ranged between 87.5% (95%CI 67.6–97.3) in MM patients and 100% in patients with NHBCL and each of the other underlying diseases at 1 M post-dose two. At 24 M post-dose two, the CMI vaccine response rate was 69.2% (38.6–90.9) in MM and 71.4% (29.0–96.3) in NHBCL patients. In the placebo group, the CMI vaccine response rate was 0.0% across underlying diseases at 1 M post-dose 2. At 24 M post-dose two, the CMI vaccine response rate ranged between 0.0% and 50.0% (1.3–98.7; *one of two HL patients*) across underlying diseases. In the RZV group, median CD4[2+] T-cell frequencies ranged between 18.3 (in NHBCL patients; interquartile range: 1.0–48.9) and 408.6 (1.0–816.3) at pre-vaccination, were highest at 1 M post-dose two across underlying diseases (3294.2 [in NHBCL patients; 2040.4–11857.2] to 21359.7 [19334.4–23385.1]), and ranged between 1691.0 (in MM patients; 579.5–3862.9) and 16573.2 (16573.2–16573.2) at 24 M post-dose two. No increases in median CD4[2+] T-cell frequencies were observed in placebo group participants with any of the underlying diseases (Figure 3(c,d), Supplementary Table 2).

### Polyfunctionality of gE-specific CD4 T-cell responses

**Figure 4.**
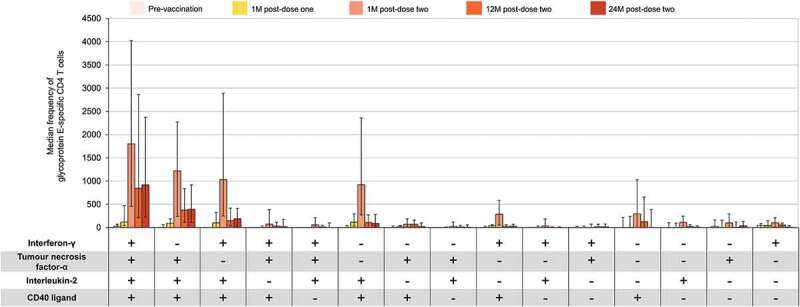
Median frequency of glycoprotein E-specific CD4 T cells expressing any combination of activation markers (adapted^†^ according-to-protocol cohort for cell-mediated immunity – RZV group only). M = month; RZV = adjuvanted recombinant zoster vaccine. Error bars depict interquartile ranges ^†^Adapted denotes that for each time point presented, the corresponding according-to-protocol cohort was used.

CD40L was the most commonly expressed marker at all time points, followed by IL-2. IFN-γ and TNF-α were usually expressed in combination with CD40L and/or IL-2. The frequencies of CD4 T cells expressing individual and combinations of activation markers were highest at 1 M post-dose two (peak of the measured response) and decreased thereafter (Figure 4).

**Figure 5.**
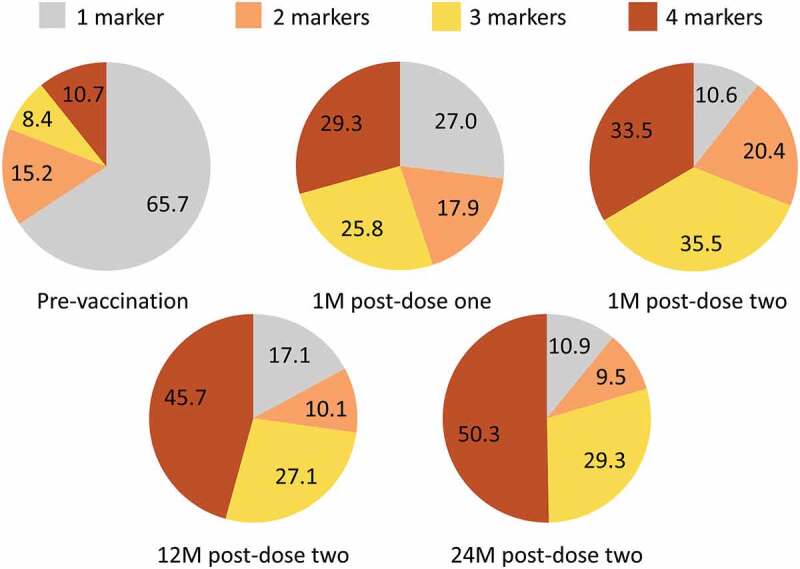
Relative mean frequencies of CD4 T cells expressing 1, 2, 3, or 4 activation markers (adapted^†^ according-to-protocol cohort for cell-mediated immunity – RZV group only). M = month; RZV = adjuvanted recombinant zoster vaccine. Data labels represent percentages of mean frequencies of CD4 T cells expressing any combination of 1, 2, 3, or 4 activation markers from: CD40 ligand; interferon-γ, interleukin-2, tumor necrosis factor-α ^†^Adapted denotes that for each time point presented, the corresponding according-to-protocol cohort was used.

Compared to pre-vaccination, the proportion of CD4 T cells expressing only one activation marker decreased from 65.7% to 27.0% and 10.6% 1 M after RZV doses one and two, respectively, while the proportion of polyfunctional CD4 T cells (expressing three or four activation markers) increased from 19.1% to 55.1% and 69.0%, respectively. The proportion of polyfunctional CD4 T cells continued to increase up to 79.6% at 24 M post-dose two (Figure 5).

### Vaccine efficacy according to underlying diseases

Vaccine efficacy against HZ was 72.4% (95% CI 54.8–83.7, *p* = .0001) in MM and 60.5% (31.0–78.2, *p* = .0006) in NHBCL patients. Vaccine efficacy was also observed for all other underlying diseases (42.5%-100%), albeit not statistically significant (Table 2).

**Table 2.** Vaccine efficacy (Poisson method) against first/only herpes zoster episode during the whole study per underlying disease (modified total vaccinated cohort)

	RZV				Placebo					
Type	N	n	Cumulative follow-up^†^ (years)	Number per 1000 person-years	N	n	Cumulative follow-up^†^ (years)	Number per 1000 person-years	Vaccine efficacy % (95% CI)	*p*-Value^‡^
Multiple myeloma	472	22	907.2	24.2	465	69	786.7	87.7	72.35 (54.76–83.71)	<0.0001
Non-Hodgkin B-cell lymphoma	237	19	438.5	43.3	244	45	410.6	109.6	60.46 (31.02–78.16)	0.0006
Non-Hodgkin T-cell lymphoma	43	1	78.9	12.7	40	5	69.2	72.3	82.45 (−56.81–99.63)	0.1633
Hodgkin lymphoma	74	5	136.9	36.5	60	7	110.2	63.5	42.50 (−110.44–85.61)	0.5028
Acute myeloid leukemia	20	0	32.2	0.0	16	3	19.8	151.4	100 (−48.86–100)	0.1105
Solid malignancies and autoimmune diseases	24	2	39.4	50.8	26	6	35.4	169.4	70.00 (−67.75–97.04)	0.2253

CI = confidence interval; N = number of participants included in each group; n = number of participants having at least one confirmed herpes zoster episode; RZV = adjuvanted recombinant zoster vaccine.

**^†^**Censored at the first occurrence of a confirmed herpes zoster episode and at the occurrence of treatment for relapse.

^‡^p-value = Two-sided exact *p*-value conditional to number of cases.

## Discussion

Robust humoral and CMI responses were elicited in participants aged 18–49 and ≥50 years, with no major differences between the two age cohorts. While humoral immune responses declined to close to baseline levels in 18–49-year-olds through 24 M post-vaccination, CMI remained high in both age cohorts. Efficacy of RZV was similar in these two age cohorts,^19^ consistent with the fact that CMI responses are believed to be the main mechanistic driver for protection against HZ.^21^ The frequencies of gE-specific CD4[2+] T cells after vaccination in autologous HSCT patients aged ≥50 years were higher than those previously reported in immunocompetent adults aged ≥50 years, using the same assay.^20^ This may be explained by the proliferation triggered post-vaccination in a not yet fully reconstituted immunological space, and thus without the classical homeostatic control. Additionally, as the data are expressed as frequencies per 10^6^ CD4 T cells, the low number of total CD4 T cells may lead to a higher proportion of gE-specific CD4[2+] T cells following vaccination early post-transplant.

**Figure 6.**
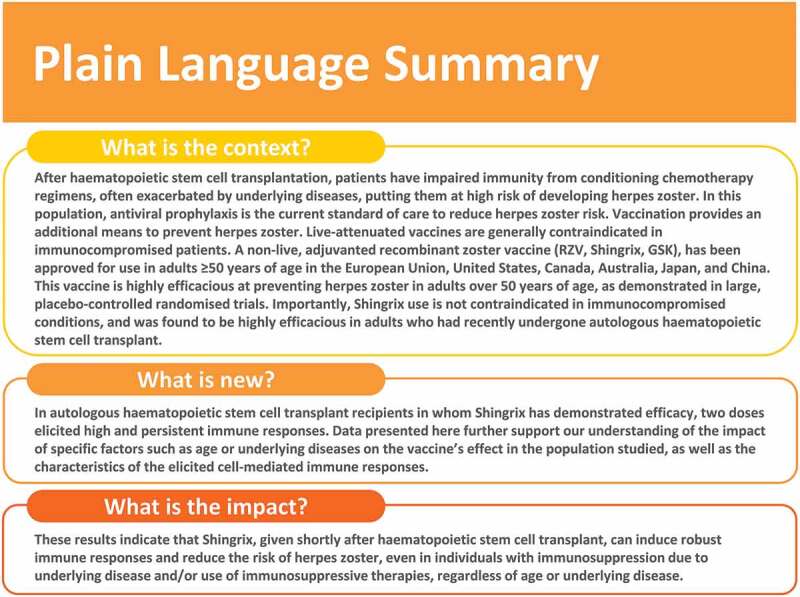
Plain language summary.

Underlying diseases appeared to have little impact on the humoral immune responses, except for NHBCL, as these patients commonly receive immunotherapeutic agents specifically targeting B cells, such as anti-CD 20 antibodies, humoral responses were expected to be low.^22^ This is consistent with the blunted post-vaccination anti-gE immune response observed in a phase I/II trial of RZV in autologous HSCT recipients, which was attributed to B-cell depletion during pre-HSCT conditioning lymphoma treatment.^18^ In contrast, underlying diseases (including NHBCL) did not appear to impact the CMI responses, which is in line with previous observations in autologous HSCT recipients.^18^ In NHBCL patients, RZV was >60% efficacious against HZ, supportive of the belief that CMI responses are the main mechanistic driver for protection against HZ.^21^

Polyfunctional CD4 T-cell responses have been shown to correlate with efficacy of vaccination against human immunodeficiency virus, tuberculosis, malaria, or melanoma.^23–26^ Cunningham et al postulated the importance of gE-specific polyfunctional CD4 T-cell responses in driving the high efficacy of RZV against HZ in adults aged ≥50 years.^20^ These findings were later confirmed in the control arm of a trial evaluating immunogenicity of RZV in previous live-attenuated zoster vaccine (ZVL, Zostavax, Merck Sharp & Dohme Corp.) recipients.^27^ In a similar evaluation, polyfunctionality was assessed in this study selecting the following four T-cell markers: IL-2, CD40L, which are the dominant activation markers shortly post-vaccination,^20^ and IFN-γ and TNF-α, which correlate best with vaccine-induced protection.^28,29^ The proportion of polyfunctional CD4 T cells increased from 1 M to 24 M post-second vaccine dose, an increase observed previously in adults aged ≥50 years.^20^ Of interest in the specific context of HSCT, the high proportion (>50%) of polyfunctional gE-specific CD4 T cells 1 M post-first vaccine dose may suggest the presence of gE-specific memory T cells in the transplanted grafts, which would be readily recalled by the first vaccine dose and further amplified by the second dose. It is unclear whether naïve or effector memory T cells predominate in the expansion phase after vaccination with ZVL, although it has been proposed that broadening of VZV-specific T-cell repertoire occurs through preferential expansion of infrequent T-cell clones, including recruitment of new specificities from the naïve repertoire.^30^ Further investigations would be required to evaluate whether this also applies to RZV.

Vaccination increased the frequency of IL-2-producing CD4 T cells. It was recently proposed that IL-2 expression at the peak of the response is required for CD4 T-cell response persistence, and this may be a key differentiator between RZV and ZVL, which may explain the observed differences in both immune responses and efficacy.^28^ ZVL induces an effector response profile dominated by IFN-ɣ, which wanes rapidly over time, while the memory T-cell profile induced by RZV, linked to IL-2 production, may explain the long-term persistence of both cellular responses and vaccine-induced protection. As in the overall study population,^19^ efficacy against HZ was observed for each underlying disease, although due to the small sample size and few incident HZ episodes, it was not statistically significant for some of these.

For establishing the optimal timing of vaccination in HSCT patients, several factors need to be considered, such as the long-term effect of conditioning regimens, post-transplant immune reconstitution and immunosuppressive regimens, and post-transplant antiviral prophylaxis. At odds with the previous observation that HSCT patients may be unable to mount a protective immune response to vaccination when given shortly after transplantation,^17^ this study demonstrated both high vaccine efficacy and strong immune responses after the two-dose RZV course initiated 50–70 days post-autologous HSCT.^19^ This is especially important to inform health care providers on timing of vaccination when protection against vaccine-preventable diseases is imperative early post-transplant such as the protection against Coronavirus Disease 2019 (COVID-19), for which treatment options and other prophylactic interventions are currently very limited. In addition, the efficacy of the evaluated RZV regimen against HZ was similar between autologous HSCT recipients who received no antiviral prophylaxis and those who received antiviral prophylaxis for up to 60 days after 1 M post-dose two,^19^ confirming the added benefit of vaccination early post-transplant. It also suggests that antiviral prophylaxis for the purpose of HZ prevention may be stopped at 6 M post-autologous HSCT, when the vaccine-elicited protection against HZ has already been achieved. While smaller studies showed that RZV (administered at a median 8–9 months post-transplant) was also well-tolerated in allogenic HSCT recipients, it was less immunogenic than in autologous HSCT recipients.^31,32^ However, administration of RZV before full immune reconstitution as well as its efficacy are yet to be evaluated in allogenic HSCT recipients.

Potential limitations of these descriptive analyses are mostly related to the small sample size and low HZ incidence in some sub-groups along with the post-hoc nature of most presented analyses, which also impact the robustness of efficacy data by underlying diseases. Details of pre-transplant conditioning treatment such as rituximab were only recorded for thirty days before transplant, and the information on the impact of specific long-lasting treatments on immune responses is incomplete. Nevertheless, the vaccine was administered 50–70 days after the autologous HSCT, when the immune system suppression was still near its maximum,^33^ and these descriptive analyses provide important insight for understanding the effect of the vaccine in this immunocompromised population. Although the follow-up period for efficacy was relatively short, it covered the first year post-transplant, prior to complete immunological recovery in some instances, when the risk of HZ is the highest.^33^

A plain language summary contextualizing the results and potential clinical research relevance and impact is presented in Figure 6.

In conclusion, two RZV doses, administered 50–70 days post-transplant, induced strong and persistent humoral and CMI responses irrespective of age, and robust CMI responses irrespective of underlying diseases in adults who had undergone autologous HSCT. Glycoprotein E-specific CD4 T-cell responses were polyfunctional, and the proportion of polyfunctional CD4 T cells increased through year two post-vaccination. Post-hoc analysis of efficacy against HZ for each underlying disease was consistent with efficacy in the overall population studied,^19^ and vaccine efficacy was also high in NHBCL patients despite the weaker humoral immune responses. RZV, currently licensed in several countries for all adults aged ≥50 years and in Europe for adults aged ≥18 years at increased risk of HZ, has the potential to represent an additional prophylactic intervention in the care of patients after autologous HSCT, at high risk for HZ.

## Supplementary Material

Supplemental Material

## Data Availability

Anonymized individual participant data and study documents can be requested for further research from www.clinicalstudydatarequest.com.

## Trademark

Shingrix is a trademark of the GSK group of companies. Zostavax is a trademark of Merck Sharp & Dohme Corp.
