# Supplementary material for: Adjuvanted recombinant zoster vaccine in adult autologous stem cell transplant recipients: polyfunctional immune responses and lessons for clinical practice
Source: Hum Vaccin Immunother. 2021 Aug 18;17(11):4144–54. doi: 10.1080/21645515.2021.1953346 (PMC8828160; doi:10.1080/21645515.2021.1953346)
Supplement: Supplemental Material [file KHVI_A_1953346_SM1625.docx]

**ZOE-HSCT study group collaborators (per alphabetical order):**

Sunil Abhyankar MD, Achilles Anagnostopoulos MD, Maria Angelopoulou MD, PhD, Mickael Aoun, MD, Ibrahim Barista MD, Leanne Berkahn MBChB, Michael J. Boeckh MD, PhD, Peter Brossart MD, Francis K. Buadi MBChB, Claude-Eric Bulabois, MD, Magda Campins MD, PhD, Guy Cantin MD, Pranatharthi Haran Chandrasekar MD, Thomas Chauncey MD, Sanjeet Singh Dadwal MD, Javier de la Serna, MD, Michael Dickinson, MBBS (hons), DMedSci, FRACP, FRCPA, Albert Esquirol Sanfeliu MD, Christelle Ferra Coll MD, PhD, Phyllis R. Flomenberg MD, Gianluca Gaidano MD, PhD, David J. Gottlieb MD, Sigal Grisariu MD, Jonathan Gutman MD, Uwe Hahn MD, Werner J. Heinz MD, Inmaculada Heras MD, PhD, Takashi Ikeda MD, PhD, Nicolas C. Issa MD, Anna M. Johnston MBBS, Meinolf Karthaus MD, Tessa Kerre MD, PhD, Alexander Kiani MD, Andreas K. Klein MD, Grzegorz Kofla MD, Irina V. Kryuchkova MD, PhD, Ching-Yuan Kuo MD, John Kuruvilla MD, Aleksey Kuvshinov Dr, Dong-Gun Lee MD, PhD, Jae Hoon Lee MD, PhD, Je-Jung Lee MD, Stéphane Lepretre MD, Albert Kwok-Wai Lie MBBS, Javier López-Jiménez MD, PhD, Johan Maertens MD, PhD, Erik W.A Marijt MD, PhD, Carmen Martínez Muñoz MD, Mariagrazia Michieli MD, Samuel T. Milliken MBBS, Noël Milpied MD, Jorge Monserrat Coll MD, Sherif Beniameen Mossad MD, John Murphy MBChB, María Belén Navarro Matilla, MD, Jan Novak, MD, PhD, Harold J. Olney, MD, CM, Raquel Oña Navarrete, MD, PhD, María Jesús Pascual Cascón MD, Andrew Peniket MD, Ganeva Penka Dr, Jaime Perez de Oteyza MD, PhD, Beata Piątkowska-Jakubas DSc, MD, David Pohlreich MD, Dimas Quiel MD, Philippe Quittet MD, Alberto Rocci MD, PhD, Scott D. Rowley MD, FACP, Waleed Sabry MD, MSc, PhD, Tommi Mikael Salmi MD, Michael J. Satlin MD, Stefan Schwartz MD, Dominik L.D. Selleslag MD, Gerda Silling MD, Sang Kyun Sohn MD, PhD, Carlos Solano, MD , PhD, Peter Staib MD, PhD, Jeff Szer MBBS, Sen Mui Tan MD, Koen Theunissen MD, Pervin Topcuoglu MD, Mikhail Uvarov MD, PhD, Carlos Vallejo Llamas MD, PhD, Fadilah S Abdul Wahid PhD, Zeynep Arzu Yegin Assoc Prof Dr, Su-Peng Yeh MD, Sze-Fai Yip MD, Sung Soo Yoon MD, PhD, Jo-Anne H. Young MD, Francesco Zaja MD, Tsila Zuckerman MD

**Supplementary Text 1 Assessment of HZ cases and estimation of vaccine efficacy**

A suspected herpes zoster (HZ) episode was defined as (1) a new rash characteristic of HZ (e.g., unilateral, dermatomal, and accompanied by pain, broadly defined to include allodynia, pruritus, or other sensations), or a vesicular rash suggestive of varicella-zoster virus (VZV) infection regardless of the distribution, and no alternative disease; or (2) clinical symptoms and/or signs suggestive of VZV infection and specific laboratory findings, such as VZV-positive culture or immune-histological staining or real-time polymerase chain reaction assay in the absence of characteristic HZ or VZV rash. Suspected HZ cases were confirmed by polymerase chain reaction or by a blinded ascertainment committee.

Vaccine efficacy was defined as 1 minus the ratio of the incidence of HZ in the vaccine group divided by the incidence in the placebo group, multiplied by 100. Vaccine efficacy was calculated using the exact inference on the relative risk conditionally to the number of cases and time at risk.

**Supplementary Text 2 Assessment of glycoprotein E-specific CD4 T cell responses**

Glycoprotein E (gE)-specific cell-mediated immunity (CMI) responses were measured using a GSK in-house assay. Peripheral blood mononuclear cells (PBMCs) were separated from heparinized blood over Ficoll-hypaque, frozen, cryopreserved and stored at -196 °C before testing.^1^ Viability of thawed PBMCs, checked prior to testing by intracellular cytokine staining, was required to be >80%.^2^ PBMCs were stimulated for 2 hours with a pool of 134 15-mer peptides overlapping by 11 (1.3 μg/mL each) spanning the entire gE ectodomain (residues 1 to 546) (Eurogentec), before 18-hour overnight incubation with brefeldin A (1μg/mL) at 37 °C. The PBMC preparation was performed by certified operators, and the certification process includes stimulation with SEB to ensure immunocompetence of separated cells. Cells were stained with a viability dye and for phenotypic surface markers (CD4), fixed, permeabilized, and stained with antibodies to CD3, CD40L, IFN-γ, TNF- α and IL-2. Cells were then washed and analyzed by flow cytometry, as previously described.^2^ An example of the employed gating strategy is provided in Supplementary Figure 1.

1. Weinberg A, Song LY, Wilkening C, Sevin A, Blais B, Louzao R, et al. Optimization and limitations of use of cryopreserved peripheral blood mononuclear cells for functional and phenotypic T-cell characterization. Clin Vaccine Immunol 2009; 16:1176-1186; doi:10.1128/cvi.00342-08
2. Moris P, van der Most R, Leroux-Roels I, Clement F, Dramé M, Hanon E, et al. H5N1 influenza vaccine formulated with AS03 A induces strong cross-reactive and polyfunctional CD4 T-cell responses. J Clin Immunol 2011; 31:443-454; doi:10.1007/s10875-010-9490-6

**Supplementary Figure 1 Example of step-by-step gating strategy used for the 7p intracellular cytokine staining assay**

**
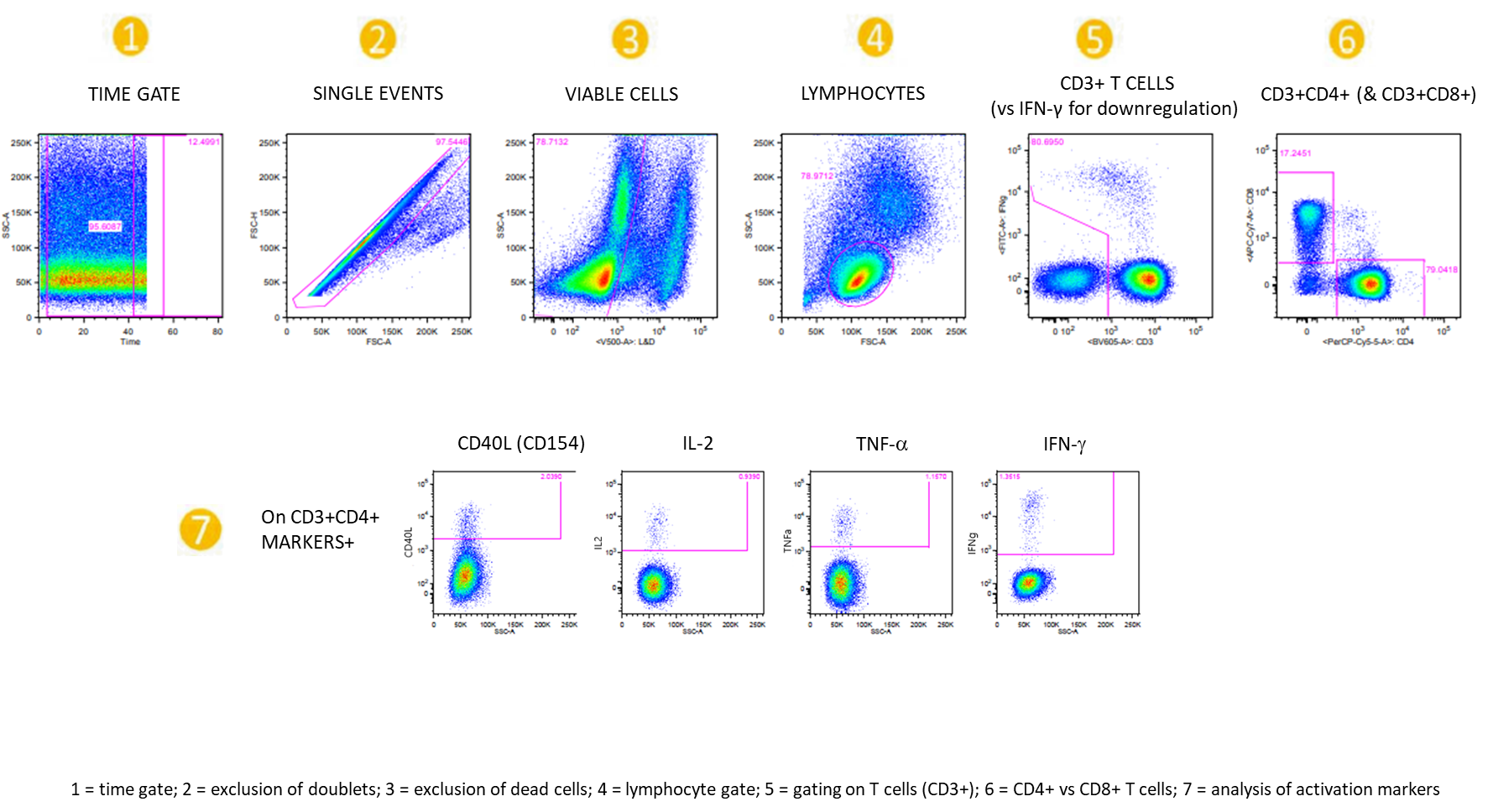
**

**Supplementary Table 1** **Anti-glycoprotein E pre-vaccination seropositivity, humoral vaccine response rate, and ELISA antibody geometric mean concentration according to age** **(****adapted**^†^ **according-to-protocol cohort for humoral immunity/persistence)**

| **Pre-vaccination seropositivity* for anti-glycoprotein E antibody** | | | | | | | | | | | | |
| --- | --- | --- | --- | --- | --- | --- | --- | --- | --- | --- | --- | --- |
|  | **18-49 years of age** | | | | | | **≥50 years of age** | | | | | |
|  | **RZV** | | | **Placebo** | | | **RZV** | | | **Placebo** | | |
|  | **N** | **n** | **% (95% CI)** | **N** | **n** | **% (95% CI)** | **N** | **n** | **% (95% CI)** | **N** | **n** | **% (95% CI)** |
|  | 26 | 26 | 100 (86.8–100) | 17 | 14 | 82.4 (56.6–96.2) | 56 | 52 | 92.9 (82.7–98.0) | 59 | 54 | 91.5 (81.3–97.2) |
| **Humoral vaccine response rate** | | | | | | | | | | | | |
|  | **18-49 years of age** | | | | | | **≥50 years of age** | | | | | |
|  | **Vaccine** | | | **Placebo** | | | **Vaccine** | | | **Placebo** | | |
| **Timing** | **N** | **n'** | **% (95% CI)** | **N** | **n’** | **% (95% CI)** | **N** | **n'** | **% (95% CI)** | **N** | **n'** | **% (95% CI)** |
| 1M post-dose one | 25 | 7 | 28.0 (12.1–49.4) | 16 | 0 | 0.0 (0.0–20.6) | 53 | 16 | 30.2 (18.3–44.3) | 55 | 0 | 0.0 (0.0–6.5) |
| 1M post-dose two | 26 | 15 | 57.7 (36.9–76.6) | 17 | 0 | 0.0 (0.0–19.5) | 56 | 40 | 71.4 (57.8–82.7) | 59 | 0 | 0.0 (0.0–6.1) |
| 12M post-dose two | 18 | 6 | 33.3 (13.3–59.0) | 11 | 1 | 9.1 (0.2–41.3) | 34 | 15 | 44.1 (27.2–62.1) | 34 | 3 | 8.8 (1.9–23.7) |
| 24M post-dose two | 13 | 3 | 23.1 (5.0–53.8) | 9 | 2 | 22.2 (2.8–60.0) | 25 | 14 | 56.0 (34.9–75.6) | 18 | 2 | 11.1 (1.4–34.7) |
| **Anti-glycoprotein E antibody geometric mean concentration (milli international units per milliliter)** | | | | | | | | | | | | |
|  | **18-49 years of age** | | | | | | **≥50 years of age** | | | | | |
|  | **RZV** | | | **Placebo** | | | **RZV** | | | **Placebo** | | |
| **Timing** | **N** | **Value (95% CI)** | | **N** | **Value (95% CI)** | | **N** | **Value (95% CI)** | | **N** | **Value (95% CI)** | |
| Pre-vaccination | 26 | 1011.0 (629.7–1623.2) | | 17 | 360.4 (190.8–680.9) | | 56 | 669.3 (460.2–973.5) | | 59 | 628.5 (435.1–907.9) | |
| 1M post-dose one | 25 | 2117.8 (1033.6–4339.4) | | 16 | 398.0 (216.7–731.0) | | 53 | 1727.7 (1123.2–2657.3) | | 55 | 613.6 (424.7–886.6) | |
| 1M post-dose two | 26 | 12523.4 (4950.7–31679.7) | | 17 | 321.2 (178.0–579.8) | | 56 | 12861.3 (7366.4–22455.0) | | 59 | 487.1 (345.6–686.6) | |
| 12M post-dose two | 19 | 3601.6 (1361.2–9529.5) | | 11 | 250.8 (104.5–602.0) | | 35 | 2977.6 (1530.1–5794.6) | | 34 | 631.1 (350.0–1137.7) | |
| 24M post-dose two | 14 | 1492.5 (466.3–4777.1) | | 9 | 351.7 (106.9–1156.7) | | 25 | 4025.0 (1597.6–10140.1) | | 19 | 638.3 (274.4–1484.9) | |

CI = confidence interval; M = month(s); N = number of participants with available results; n = number of participants with concentration ≥97 milli international units per milliliter; n’, number of participants with a humoral vaccine response; ELISA = enzyme-linked immunosorbent assay; RZV = adjuvanted recombinant zoster vaccine

^†^Adapted denotes that for each time point presented, the corresponding per protocol cohort was used

*The seropositivity assay cut-off was 97 milli-International Units per milliliter

**Supplementary Table 2 Cell-mediated immunity vaccine response rate and glycoprotein E-specific CD4[2+] T-cell frequency according to age and underlying diseases (adapted^†^ according-to-protocol cohort for cell-mediated immunity/persistence)**

|  | **Cell-mediated immunity vaccine response rate** | | | | | | **Frequency of CD4[2+] T cells** | | | |
| --- | --- | --- | --- | --- | --- | --- | --- | --- | --- | --- |
|  | **RZV** | | | **Placebo** | | | **RZV** | | **Placebo** | |
| **Timing** | **N** | **n** | **%**  **(95% CI)** | **N** | **n** | **%**  **(95% CI)** | **N** | **Median**  **(Min [Q1; Q3] Max)** | **N** | **Median**  **(Min [Q1; Q3] Max)** |
| **Analyses according to age** | | | | | | | | | | |
| 18-49 years of age | | | | | | | | | | |
| Pre-vaccination | .. | .. | .. | .. | .. |  | 16 | 77.9 (1.0[9.7;213.9]816.3) | 10 | 110.0 (1.0[1.0;132.6]356.1) |
| 1M post-dose one | 12 | 9 | 75.0  (42.8-94.5) | 9 | 0 | 0.0  (0.0-33.6) | 15 | 1411.4 (129.5[483.2;2180.0]10825.0) | 12 | 1.2 (1.0[1.0;103.2]288.6) |
| 1M post-dose two | 14 | 14 | 100  (76.8-100) | 9 | 0 | 0.0  (0.0-33.6) | 16 | 12365.5 (1.0[3591.1;21624.6]26004.7) | 12 | 48.3 (1.0[1.0;187.6]556.9) |
| 12M post-dose two | 10 | 9 | 90.0  (55.5-99.7) | 5 | 0 | 0.0  (0.0-52.2) | 13 | 4872.2 (136.8[2133.7;6709.3]13292.0) | 6 | 57.4 (1.0[1.0;127.5]303.7) |
| 24M post-dose two | 7 | 7 | 100  (59.0-100) | 5 | 1 | 20.0  (0.5-71.6) | 10 | 3466.0 (86.0[1969.9;5087.5]16573.2) | 6 | 186.6 (1.0[91.4;243.2]1050.8) |
| ≥50 years of age | | | | | | | | | | |
| Pre-vaccination | .. | .. | .. | .. | .. | .. | 31 | 34.0 (1.0[1.0;185.2]2469.7) | 37 | 43.8 (1.0[1.0;193.5]874.9) |
| 1M post-dose one | 29 | 10 | 34.5  (17.9-54.3) | 34 | 0 | 0.0  (0.0-10.3) | 34 | 382.3 (1.0[127.8;1085.3]14121.1) | 38 | 31.6 (1.0[1.0;155.2]597.0) |
| 1M post-dose two | 28 | 25 | 89.3  (71.8-97.7) | 32 | 0 | 0.0  (0.0-10.9) | 35 | 3294.2 (1.0[1017.1;11135.7]73143.3) | 36 | 84.8 (1.0[1.0;171.0]408.7) |
| 12M post-dose two | 17 | 10 | 58.8  (32.9-81.6) | 21 | 2 | 9.5  (1.2-30.4) | 19 | 1152.5 (1.0[453.8;2463.4]17462.0) | 23 | 80.9 (1.0[1.0;173.2]722.0) |
| 24M post-dose two | 17 | 10 | 58.8  (32.9-81.6) | 11 | 1 | 9.1  (0.2-41.3) | 20 | 1519.6 (52.0[281.4;3155.0]26020.4) | 12 | 65.3 (1.0[1.0;244.0]642.5) |
| **Analyses according to underlying diseases** | | | | | | | | | | |
| Multiple myeloma | | | | | | | | | | |
| Pre-vaccination | .. | .. | .. | .. | .. | .. | 26 | 57.0 (1.0[1.0;207.4]2469.7) | 28 | 81.7 (1.0[1.0;195.1]356.1) |
| 1M post-dose one | 25 | 12 | 48.0  (27.8-68.7) | 25 | 0 | 0.0  (0.0-13.7) | 28 | 470.4 (1.0[92.0;1598.8]14121.1) | 28 | 44.8 (1.0[1.0;166.0]439.4) |
| 1M post-dose two | 24 | 21 | 87.5  (67.6-97.3) | 24 | 0 | 0.0  (0.0-14.2) | 29 | 4110.1 (1.0[1017.1;9575.4]73143.3) | 27 | 118.3 (1.0[15.0;195.7]556.9) |
| 12M post-dose two | 15 | 9 | 60.0  (32.3-83.7) | 15 | 1 | 6.7  (0.2-31.9) | 16 | 1209.2 (1.0[476.4;2788.5]17462.0) | 15 | 80.9 (1.0[1.0;229.3]682.1) |
| 24M post-dose two | 13 | 9 | 69.2  (38.6-90.9) | 10 | 1 | 10.0  (0.3-44.5) | 16 | 1691.0 (94.4[579.5;3862.9]26020.4) | 10 | 111.8 (1.0[1.0;243.2]642.5) |
| Non-Hodgkin B-cell lymphoma | | | | | | | | | | |
| Pre-vaccination | .. | .. | .. | .. | .. | .. | 13 | 18.3 (1.0[1.0;48.9]338.3) | 9 | 39.0 (1.0[1.0;116.2]210.0) |
| 1M post-dose one | 11 | 3 | 27.3  (6.0-61.0) | 9 | 0 | 0.0  (0.0-33.6) | 14 | 410.8 (129.5[285.7;1061.9]2916.4) | 12 | 31.6 (1.0[1.0;106.7]226.8) |
| 1M post-dose two | 12 | 12 | 100  (73.5-100) | 7 | 0 | 0.0  (0.0-41.0) | 15 | 3294.2 (1.0[2040.4;11857.2]26004.7) | 10 | 43.6 (1.0[1.0;106.7]371.3) |
| 12M post-dose two | 8 | 6 | 75.0  (34.9-96.8) | 5 | 1 | 20.0  (0.5-71.6) | 10 | 1636.3 (136.8[609.7;4319.6]7502.6) | 8 | 49.1 (1.0[1.0;148.8]722.0) |
| 24M post-dose two | 7 | 5 | 71.4  (29.0-96.3) | 3 | 0 | 0.0  (0.0-70.8) | 8 | 1696.8 (52.0[73.8;2965.2]5087.5) | 5 | 102.7 (1.0[1.0;230.2]257.7) |
| Non-Hodgkin T-cell lymphoma | | | | | | | | | | |
| Pre-vaccination | .. | .. | .. | .. | .. | .. | 3 | 353.6 (106.3[106.3;556.6]556.6) | 3 | 65.0 (1.0[1.0;874.9]874.9) |
| 1M post-dose one | 1 | 1 | 100  (2.5-100) | 3 | 0 | 0.0  (0.0-70.8) | 1 | 1323.5 (1323.5[1323.5;1323.5]1323.5) | 3 | 119.2 (1.0[1.0;597.0]597.0) |
| 1M post-dose two | 2 | 2 | 100  (15.8-100) | 3 | 0 | 0.0  (0.0-70.8) | 2 | 18717.7 (16149.7[16149.7;21285.6]21285.6) | 3 | 272.5 (1.0[1.0;316.0]316.0) |
| 12M post-dose two | 1 | 1 | 100  (2.5-100) | 1 | 0 | 0.0  (0.0-97.5) | 1 | 11333.3 (11333.3[11333.3;11333.3]11333.3) | 1 | 101.3 (101.3[101.3;101.3]101.3) |
| 24M post-dose two | 2 | 1 | 50.0  (1.3-98.7) | 1 | 0 | 0.0  (0.0-97.5) | 2 | 2044.2 (455.2[455.2;3633.2]3633.2) | 1 | 1.0 (1.0[1.0;1.0]1.0) |
| Hodgkin lymphoma | | | | | | | | | | |
| Pre-vaccination | .. | .. | .. | .. | .. | .. | ..1 | 183.2 (183.2[183.2;183.2]183.2) | 2 | 113.4 (112.8[112.8;113.9]113.9) |
| 1M post-dose one | 1 | 1 | 100  (2.5-100) | 2 | 0 | 0.0  (0.0-84.2) | 1 | 781.7 (781.7[781.7;781.7]781.7) | 3 | 1.0 (1.0[1.0;1.4]1.4) |
| 1M post-dose two | 0 | - | - | 2 | 0 | 0.0  (0.0-84.2) | 0 |  | 3 | 1.0 (1.0[1.0;1.0]1.0) |
| 12M post-dose two | 1 | 1 | 100  (2.5-100) | 2 | 0 | 0.0  (0.0-84.2) | 1 | 4872.2 (4872.2[4872.2;4872.2]4872.2) | 2 | 57.4 (1.0[1.0;113.8]113.8) |
| 24M post-dose two | 1 | 1 | 100  (2.5-100) | 2 | 1 | 50.0  (1.3-98.7) | 1 | 3298.8 (3298.8[3298.8;3298.8]3298.8) | 2 | 571.1 (91.4[91.4;1050.8]1050.8) |
| Acute myeloid leukemia | | | | | | | | | | |
| Pre-vaccination | .. | .. | .. | .. | .. | .. | ..2 | 114.2 (1.0[1.0;227.3]227.3) | 2 | 66.8 (1.0[1.0;132.6]132.6) |
| 1M post-dose one | 1 | 1 | 100  (2.5-100) | 2 | 0 | 0.0  (0.0-84.2) | 1 | 1411.4 (1411.4[1411.4;1411.4]1411.4) | 2 | 44.1 (1.0[1.0;87.2]87.2) |
| 1M post-dose two | 2 | 2 | 100  (15.8-100) | 2 | 0 | 0.0  (0.0-84.2) | 2 | 21359.7 (19334.4[19334.4;23385.1]23385.1) | 2 | 5.6 (1.0[1.0;10.2]10.2) |
| 12M post-dose two | 1 | 1 | 100  (2.5-100) | 1 | 0 | 0.0  (0.0-97.5) | 1 | 12648.8 (12648.8[12648.8;12648.8]12648.8) | 1 | 1.0 (1.0[1.0;1.0]1.0) |
| 24M post-dose two | 1 | 1 | 100  (2.5-100) | 0 | - | - | 1 | 16573.2 (16573.2[16573.2;16573.2]16573.2) | 0 |  |
| Solid malignancies and others | | | | | | | | | | |
| Pre-vaccination | .. | .. | .. | .. | .. | .. | 2 | 408.6 (1.0[1.0;816.3]816.3) | 3 | 1.0 (1.0[1.0;1.0]1.0) |
| 1M post-dose one | 2 | 1 | 50.0  (1.3-98.7) | 2 | 0 | 0.0  (0.0-84.2) | 4 | 2944.7 (483.2[531.0;8067.9]10825.0) | 2 | 31.7 (1.0[1.0;62.3]62.3) |
| 1M post-dose two | 2 | 2 | 100  (15.8-100) | 3 | 0 | 0.0  (0.0-70.8) | 3 | 11506.1 (11135.7[11135.7;21963.7]21963.7) | 3 | 16.0 (1.0[1.0;59.2]59.2) |
| 12M post-dose two | 1 | 1 | 100  (2.5-100) | 2 | 0 | 0.0  (0.0-84.2) | 3 | 6709.3 (2463.4[2463.4;13292.0]13292.0) | 2 | 187.3 (124.6[124.6;250.0]250.0) |
| 24M post-dose two | 0 | - | - | 0 | - | - | 2 | 6589.6 (3187.1[3187.1;9992.1]9992.1) | 0 |  |

CD4[2+] T cells = CD4 T cells expressing ≥2 of the following activation markers: interferon-γ, interleukin-2, tumor necrosis factor-α, and cluster of differentiation 40 ligand; CI = confidence interval; M = month(s); N = number of participants with available results; n = number of participants with a cell-mediated immunity vaccine response; Q1, Q3 = first and third quartiles; RZV = adjuvanted recombinant zoster vaccine

^†^Adapted denotes that for each time point presented, the corresponding per-protocol cohort was used

**Supplementary Table 3 Anti-glycoprotein E pre-vaccination seropositivity, humoral vaccine response rate, and ELISA geometric mean concentration according to underlying diseases** **(modified total vaccinated cohort)**

|  | **Pre-vaccination seropositivity* for anti-glycoprotein E antibody** | | | | | | | | |
| --- | --- | --- | --- | --- | --- | --- | --- | --- | --- |
|  | **Vaccine** | | | | **Placebo** | | | | |
|  | **N** | **n** | **% (95% CI)** | | **N** | | **n** | **% (95% CI)** | |
|  | | | | | | | | | |
| Multiple myeloma | 468 | 420 | 89.7 (86.6–92.3) | | 459 | | 415 | 90.4 (87.3–92.9) | |
| Non-Hodgkin B-cell lymphoma | 235 | 231 | 98.3 (95.7–99.5) | | 242 | | 238 | 98.3 (95.8–99.5) | |
| Non-Hodgkin T-cell lymphoma | 42 | 42 | 100 (91.6–100) | | 39 | | 37 | 94.9 (82.7–99.4) | |
| Hodgkin lymphoma | 74 | 72 | 97.3 (90.6–99.7) | | 60 | | 56 | 93.3 (83.8–98.2) | |
| Acute myeloid leukemia | 20 | 20 | 100 (83.2–100) | | 16 | | 16 | 100 (79.4–100) | |
| Other diseases* | 24 | 23 | 95.8 (78.9–99.9) | | 25 | | 25 | 100 (86.3–100) | |
|  | **Humoral vaccine response rate at 1M post-dose two** | | | | | | | | |
|  | **Vaccine** | | | | **Placebo** | | | | |
|  | **N** | **n'** | **Value (95% CI)** | | **N** | | **n'** | **Value (95% CI)** | |
|  | | | | | | | | | |
| Multiple myeloma | 440 | 399 | 90.7 (87.6–93.2) | | 437 | | 13 | 3.0 (1.6–5.0) | |
| Non-Hodgkin B-Cell lymphoma | 226 | 32 | 14.2 (9.9–19.4) | | 224 | | 0 | 0.0 (0.0–1.6) | |
| Non-Hodgkin T-Cell lymphoma | 40 | 28 | 70.0 (53.5–83.4) | | 35 | | 0 | 0.0 (0.0–10.0) | |
| Hodgkin lymphoma | 68 | 51 | 75.0 (63.0–84.7) | | 59 | | 1 | 1.7 (0.0–9.1) | |
| Acute myeloid leukemia | 20 | 17 | 85.0 (62.1–96.8) | | 16 | | 0 | 0.0 (0.0–20.6) | |
| Solid malignancies and others | 23 | 16 | 69.6 (47.1–86.8) | | 24 | | 0 | 0.0 (0.0–14.2) | |
|  | **Anti-glycoprotein E antibody geometric mean concentration (milli international units per millilitre)** | | | | | | | | |
|  | **Vaccine** | | | | **Placebo** | | | | |
|  | **N** | | **Value (95% CI)** | | **N** | | | **Value (95% CI)** | |
| Multiple myeloma | | | | | | | | | |
| Pre-vaccination | 468 | | 470.6 (415.6–532.8) | | 459 | | | 461.9 (410.9–519.3) | |
| 1M post-dose two | 444 | | 32549.3 (27585.8–38405.7) | | 440 | | | 361.7 (318.4–410.8) | |
| Non-Hodgkin B-Cell lymphoma | | | |  | |  | | |  |
| Pre-vaccination | 235 | | 822.7 (728.2–929.4) | | 242 | | | 843.4 (748.8–950.0) | |
| 1M post-dose two | 227 | | 1378.4 (1124.4–1689.8) | | 226 | | | 761.2 (663.8–872.8) | |
| Non-Hodgkin T-Cell lymphoma | | | |  | |  | | |  |
| Pre-vaccination | 42 | | 1221.7 (853.2–1749.2) | | 39 | | | 827.9 (591.0–1159.7) | |
| 1M post-dose two | 40 | | 11052.4 (6179.4–19768.5) | | 36 | | | 711.5 (478.1–1059.1) | |
| Hodgkin lymphoma | | | |  | |  | | |  |
| Pre-vaccination | 74 | | 1114.9 (829.0–1499.5) | | 60 | | | 882.7 (635.1–1226.9) | |
| 1M post-dose two | 68 | | 22744.4 (13849.0–37353.5) | | 59 | | | 829.3 (589.8–1166.1) | |
| Acute myeloid leukemia | | | |  | |  | | |  |
| Pre-vaccination | 20 | | 759.2 (519.7–1109.1) | | 16 | | | 1082.0 (676.0–1732.0) | |
| 1M post-dose two | 20 | | 19597.6 (9458.0–40607.5) | | 16 | | | 759.1 (451.0–1277.5) | |
| Solid malignancies and others |  | |  | |  | | |  | |
| Pre-vaccination | 24 | | 735.6 (480.5–1126.1) | | 25 | | | 1047.5 (686.7–1597.9) | |
| 1M post-dose two | 23 | | 21120.6 (8305.6–53708.3) | | 25 | | | 808.5 (529.4–1234.7) | |

CI = confidence interval; M = month; N = number of participants with available results; n = number of participants with concentration ≥97 milli international units per milliliter; n’, number of participants with a humoral vaccine response; ELISA = enzyme-linked immunosorbent assay; RZV = adjuvanted recombinant zoster vaccine

*The seropositivity assay cut-off was 97 milli-International Units per milliliter
